# Creating access to SARS-CoV-2 screening and testing through community-based COVID-19 case-finding, observations from cross-sectional studies in Lesotho and Zambia

**DOI:** 10.1186/s12889-023-16306-2

**Published:** 2023-07-24

**Authors:** Eveline Klinkenberg, Bulemba Katende, Maria Ruperez, Moniek Bresser, Bxyn Kangololo, Justin Bwalya, Rahel M. Erhardt, Ab Schaap, Nkatya Kasese, Thomas Gatchie, Sian Floyd, ‘Mota J. ‘Mota, Helen Ayles, Kwame Shanaube, Klaus Reither

**Affiliations:** 1 London School of Hygiene & Tropical Medicine (LSHTM), London, UK; 2 Department of Global Health, Amsterdam University Medical Centers, Amsterdam, The Netherlands; 3 SolidarMed, Partnerships for Health, Maseru, Lesotho; 4 Swiss Tropical and Public Health Institute, Allschwil, Switzerland; 5 University of Basel, Basel, Switzerland; 6 Zambart, University of Zambia School of Public Health, Lusaka, Zambia

**Keywords:** COVID-19, Community testing, Lesotho, Zambia, SARS-CoV-2

## Abstract

**Background:**

The health impact of the COVID-19 pandemic largely depends on the ability of the healthcare systems to develop effective and adaptable preparedness and mitigation strategies. A collaborative initiative (BRCCH-EDCTP COVID-19 Initiative) was set up between Lesotho and Zambia early on in the pandemic, to jointly conduct a project to investigate creating access to SARS-CoV-2 screening and testing through community-based COVID-19 case-finding.

**Methods:**

Two different community case-finding strategies were deployed. In Lesotho, an approach was implemented whereby a community (village) health worker screened community members at their home or during community gatherings for COVID-19 signs and symptoms. All community members who screened positive were then offered SARS-CoV-2 testing. In Zambia, so-called community hubs, staffed by community health care workers, were set up at different locations in the community for people to walk in and get tested for SARS-CoV-2. Hubs changed location from week-to-week and targeted transmission hotspots. All persons visiting the hubs were offered testing for SARS-CoV-2 irrespective of self-reported signs and symptoms of COVID-19 though information was collected on occurrence of these. Testing in both approaches was done using SARS-CoV-2 rapid antigen tests.

**Results:**

Setting up testing in the community setting was feasible in both countries. In Lesotho in the village health worker approach, over a period of 46 weeks, 7221 persons were screened, and 49 (11.4%) SARS-COV-2 cases identified among 428 COVID-19 screen positive participants. In the community hubs among 3150 people tested, 166 (5.3%) SARS-CoV-2 cases were identified in a period of 26 weeks. From the community hubs approach, where all seen were offered COVID-19 testing it was learned that people screening positive for COVID-19 signs and symptoms were more likely to test SARS-COV-2 positive, especially those reporting classic COVID-19 symptoms like loss of sense/smell for a short period of time (1–3 days).

**Conclusions:**

In conclusion, in this project we learned that implementing COVID-19 screening and testing by lay health workers in the community is possible. Characteristics of the population screened, tested, and identified to have SARS-CoV-2 are described to help guide development of future testing strategies.

**Supplementary Information:**

The online version contains supplementary material available at 10.1186/s12889-023-16306-2.

## Introduction

The COVID-19 pandemic has caused a global public health emergency at unprecedented scale [1, 2]. In sub-Saharan Africa, till the end of 2022 over 9 million cases were notified with approximately 175,000 deaths [3]. The health impact of such a pandemic largely depends on the ability of the healthcare system to develop effective and adaptable preparedness and mitigation strategies, including basic preventative measures, regulations for isolation and quarantine, intensified surveillance, tracing, and treatment and care [4, 5]. These strategies should be designed to protect healthcare workers, community members, essential health care services, and should offer the best possible care to COVID-19 patients.

Screening and testing strategies are part of a comprehensive control package. In several African countries, community screening and testing strategies have been implemented building upon existing community health worker programs [6, 7]. Such a community health workers approach has yielded positive results in creating access to screening and testing for other diseases at the community level [6]. In South Africa, a community-based COVID-19 response was implemented at the very beginning of the pandemic as part of the epidemic response. The aim was to provide, on the one hand, screening, and testing services to the community and on the other hand an opportunity for direct surveillance [1].

Like other African countries the Lesotho and Zambian health systems were also negatively impacted by the COVID-19 pandemic [7–9]. Access to basic health care services including access to COVID-19 screening and testing were substantially restricted due to various factors such as inadequate number of health care workers, lack of personal protective equipment, fear of contracting SARS-CoV-2, and transport restrictions [5]. Lesotho has reported 34,490 cases and 706 deaths [10] since the beginning of the epidemic and Zambia 333,746 cases and 4019 deaths [11].

A collaborative project (BRCCH-EDCTP COVID-19 Initiative) was set up between researchers and Ministries of Health in Lesotho and Zambia to jointly investigate community based approaches for SARS-COV-2 screening and testing. In this paper, we discuss findings from the implementation of two community-based approaches for COVID-19 screening and testing and, characteristics of the population attracted by these two approaches.

## Methods

### Design

Using a cross-sectional design, we implemented two different community-based approaches for COVID-19 screening and testing in Zambia and Lesotho. In Lesotho, screening and testing was performed by village healthcare workers (VHW) at people’s home or at community gatherings. In Zambia, screening and testing was conducted at community hubs by community health workers.

### Setting

**Fig. 1:**
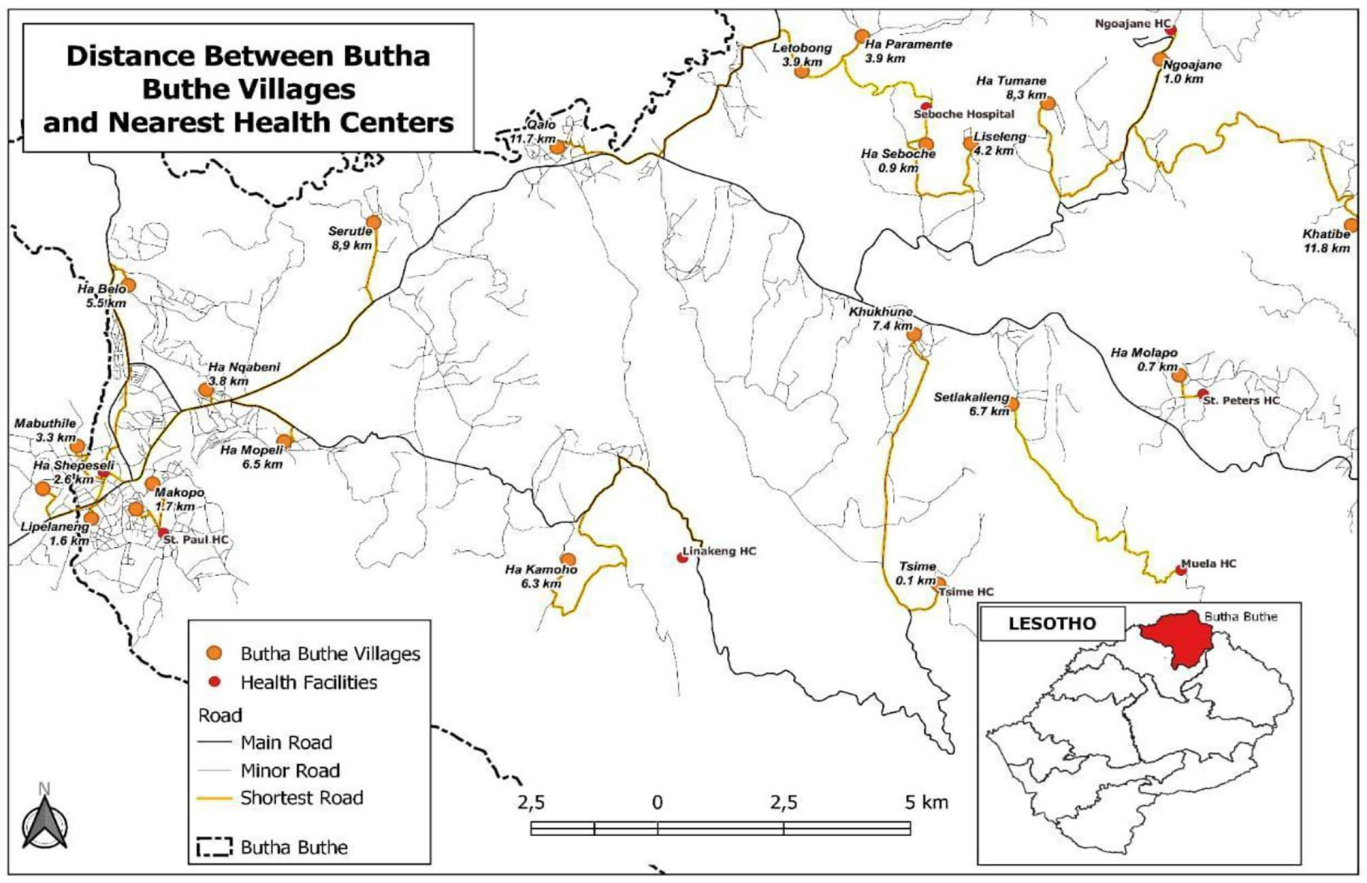
Map of Butha-Buthe district showing the location of the health facilities and villages included in the study

In Lesotho, the project was implemented in selected peri-urban and rural villages in the Butha-Buthe district. Butha-Buthe is a small district of 1,767 square km located in northern Lesotho with an estimated total population of 118,242 people of which more than 75 per cent are living in rural settings [5]. The health services in the district are delivered through a network of 10 clinics and 2 hospitals supported by a cadre of VHWs. The district had an estimated HIV prevalence of 21.2% in 2017 [4]. TB prevalence in Lesotho is estimated at ~ 0.5% in per-urban and ~ 0.7% in rural areas [12]. A total of 23 villages (9 peri-urban and 14 rural) with an estimated combined population of 40,303 [5] were selected in collaboration with the District Health Management Team (DHMT) out of a total of 2,913 villages in the district. Villages selected had high COVID-19 transmission during the December 2020 to February 2021 COVID-19 wave. Figure 1 shows the location of the selected villages and the health centres in the district.

**Fig. 2:**
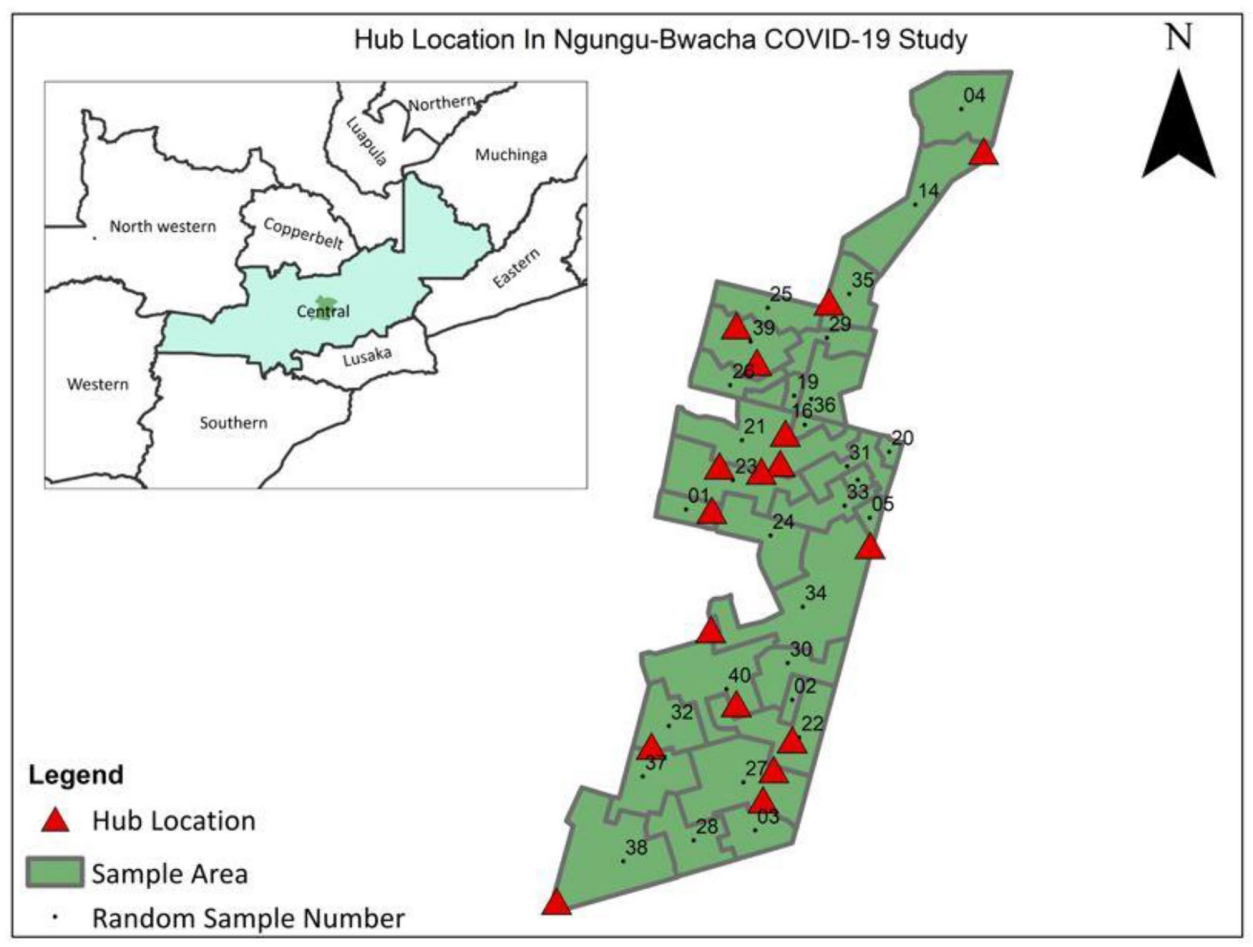
Map of the Zambia study community and it location within central province indicating location of hubs

In Zambia, the project was conducted in a peri-urban community in the Kabwe district. This community was selected as it was part of an ongoing study to gain rapid evidence on COVID-19 in an African setting. Kabwe district is a middle to high density urban area located about 15 kms north-west of Kabwe town centre in the Central Province of Zambia. This community has been previously characterised as a mixed economy typical of other Zambian peri-urban communities [13]. The total population was estimated to be 28,000 individuals, of whom around 17,000 (60%) were aged ≥ 15 years, living in approximately 5,300 households, with an average household size around 5.3 persons. HIV prevalence in the community was approximately 15% and TB prevalence was estimated to be in the range of 0.5-1% in 2017 [13]. Figure 2 shows the Zambia community indicating the different locations of the hubs.

### Design of the community-based screening and testing approaches

Table 1 provides a methodological summary of the two different approaches.

**Table 1:** Methodological summary of the two community approaches implemented

	Community hubs	Village Health Worker
Country	Zambia	Lesotho
Setting	Peri-urban (4 rotating hubs)	Rural (14)/peri-urban (9)
Time period	**26 weeks** (Week 18–43 in 2021)	**46 Weeks** (Weeks 31–52 in 2021; 1–25 2022)
Staffing	Nurses/lay HCW 2 per hub/4 hubs	24 VHWs in 23 villages/ 1 outreach nurse
Target population	Walk-ins ≥ 15 years	≥ 18 years
Coverage	Each hub 3,000 to 4,000 people - changing location	23 villages – total target population ~ 40,303
Location of screening, sample collection & testing	Hub	VHW at Home/Household/Community Gathering
Data collection Tool	Tablets linked to dedicated data management system designed for the project	Tablets linked to data management software as well as paper tools
COVID-19 screening algorithm	COVID-19 screen positive if yes to any of 5 items below: (1) fever (physically measured > 38 °C or self-reported fever in the last 5 days; (2) Any (dry) cough; (3) New shortness of breath; (4) Sudden loss of taste and/or smell (anosmia and/or ageusia); (5) HH contact of a confirmed COVID-19 case	COVID-19 screen positive if yes to any of 13 items below: reported (1) Cough of any duration; (2) fever; (3) Pronounced tiredness (4) Shortness of breath (5) Sore throat 6. Muscle or body pain 7. Diarrhoea 8. Loss of taste/smell 9. Weight loss 10. Night sweats; 11. Skin Rash, 12. Chest pain, 13. Contact with a confirmed COVID19 case
Group tested for SARS-CoV-2	All those attending the hub for health services, irrespective of reporting of COVID-19 signs and/or symptoms	COVID19 screen positive (symptomatic or HH contact)
SARS-CoV-2 test used	Antigen rapid diagnostic test (Panbio™ Ag-RDT) performed by HCWs	Antigen rapid diagnostic test (SD Biosensor Standard Q) performed by VHWs

#### Village health workers (Lesotho)

The VHW approach was implemented from the 4th of August 2021 to the 15th of June 2022 in Lesotho. In the selected villages, a VHW screened and tested community members at the participant or the VHW home or during community gatherings. VHWs worked in close collaboration with the Butha Buthe DHMT and the village chiefs from whom they received information on households with possible COVID-19 cases. Within villages, they visited those households for screening and testing. Additionally, community members who were sensitised through the village chief could call or walk to the VHW home to receive COVID-19 services when needed. Participants, who had to be at least 18 years of age, were considered screen positive for COVID-19 if they, at the time of the visit/screening, reported a history of close contact with a COVID-19 case and/or at least one of the following symptoms for any duration: cough, fever, pronounced tiredness, shortness of breath, sore throat, muscle or body pain, diarrhoea, loss of taste/smell, weight loss, night sweat, skin rash, or chest pain. We defined close contact with confirmed or probable COVID-19 case as a contact within one meter for more than 15 min or direct physical contact with a probable or confirmed COVID-19 case or direct care for a patient with probable or confirmed COVID-19 disease without using proper personal protective equipment (PPE). All screened positive were offered a COVID-19 test.

We relied on community health workers trained as VHW by the Lesotho Ministry of Health (MoH). VHWs were recruited by the project from the MoH pool using specific criteria such as ability to read and write, ability to use an electronic device such as a tablet. All recruited VHWs for the project were trained on study procedures such as basic COVID-19 knowledge, COVID-19 sample collection and processing using rapid diagnostic tests, use of a COVID-19 application developed for data collection and infection prevention and control techniques. A professional nurse supervised all the VHWs during the study period both through remote supervision as well as onsite visits.

The professional nurse was employed full-time by the project and received full salary while the VHWs continued with their assignments for the MoH and received a monthly stipend from the project for the additional work. All staff involved in the study received PPE: face mask, gowns, gloves, sanitiser etc.), symptomatic patients were provided with a surgical face mask.

#### Community hub (Zambia)

A community hub approach was designed and implemented in Zambia to create access to COVID-19 screening and testing in the community. So called community hubs were set up at different locations in the community for community members to walk in and get tested for SARS-CoV-2. From 4th May to 29th October 2021, a total of 4 hubs were operational. Location of hubs was decided in consultation with the Community Advisory Board (CAB) and targeted areas perceived to have higher transmission risk, i.e., a hot spot, community intersection or areas where many people pass or conduct community activities. Hubs changed location on weekly basis though depending on attendance, this period could be shortened or extended. Each hub served an estimated 3,000 to 4,000 people. Community members were sensitised using different community engagement approaches like drama or by loudspeaker to inform them about location of hubs and services available.

Services at the hubs targeted individuals aged 15 years and above. Upon arrival at the hub and, after consent taking, all participants were administered a simple questionnaire collecting information on socio-demographics characteristics and clinical information. Participants were also asked about COVID-19 signs and symptoms and whether they had a (known) history of contact with a confirmed COVID-19 case. In Zambia the following COVID-19 signs and symptoms were inquired about: fever (physically measured > 38 °C or self-reported fever in the last 5 days), (dry) cough, new episode of shortness of breath, sudden loss of taste and/or smell (anosmia and/or ageusia). Following the questionnaire all participants were offered COVID-19 testing regardless of reporting of signs and symptoms. Besides COVID-19 testing, HIV testing services were also offered at the hubs. In addition, all participants reporting signs and symptoms suggestive for TB were referred for TB testing. All participants identified with COVID-19 and newly diagnosed with HIV or TB were referred for appropriate care taking following national guidelines. Hubs were staffed by a nurse and community health workers who were recruited by the project from the local community, both worked full time in the hubs for the duration of the project. PPE was provided to all study staff members of the hubs, i.e. mask, sanitizer, apron and community members coming for testing were asked to wear masks as per guidelines at the time for public places. Masks were also available at the hubs free of charge.

#### Testing for SARS-CoV-2 infection

In the VHW approach, the VHW collected a nasopharyngeal swab and processed it using the Standard Q COVID-19 rapid diagnostic test (SD Biosensor, Republic of Korea). For invalid results, the test was repeated using a new sample. Both the sample collection and testing process were done at well-ventilated places agreed upon by the study participant and the VHW.

In the community hubs, a nasal swab was collected from individuals attending the hubs and tested on the spot for COVID-19 using the Panbio™ Ag-RDT (Abbott Diagnostic GmbH, Jena, Germany) following the manufacturer’s instructions for use. If the result was invalid, the test was repeated with the remainder of the sample or with a new sample. Sample collection and testing process were done at a well-ventilated location in the hub.

##### Clinical management

Study participants who tested positive and those who tested negative but had symptoms in the VHWs approach were advised on self-isolation. Symptomatic participants with a negative result were followed up within 48 h and retested if they still reported symptoms. In Zambia following national guidance, only those testing positive were advised to self-isolate at home.

### Data Collection and Analysis

Data were collected in both countries using electronic data collection tools incorporated into an electronic device (tablet). Due to technical challenges with the application, Lesotho switched from electronic data collection to paper-based data collection during the project. For every participant enrolled, in both countries, the following data were collected: age, sex, history of contact with a confirmed or probable COVID-19 case (Lesotho) or household contact (Zambia), COVID-19 signs and symptoms (including duration thereof), type of swab collected, type of tests done, and test results. In addition, TB status, whether or not the participant had a previous history of TB treatment was obtained. Both countries have a high TB burden and as COVID also affects the lungs we explored TB status as potential risk factor.

The data collected was analysed using STATA Version 15 (Zambia) and 16.1 (Lesotho). Demographics of study participants are presented using frequency tables, mean, standard deviation and 95% confidence interval (CI) for normally distributed data or median and interquartile range for skewed data. A p-value of < 0.05 was considered significant.

## Results

### Screening and enrolment

**Fig. 3:**
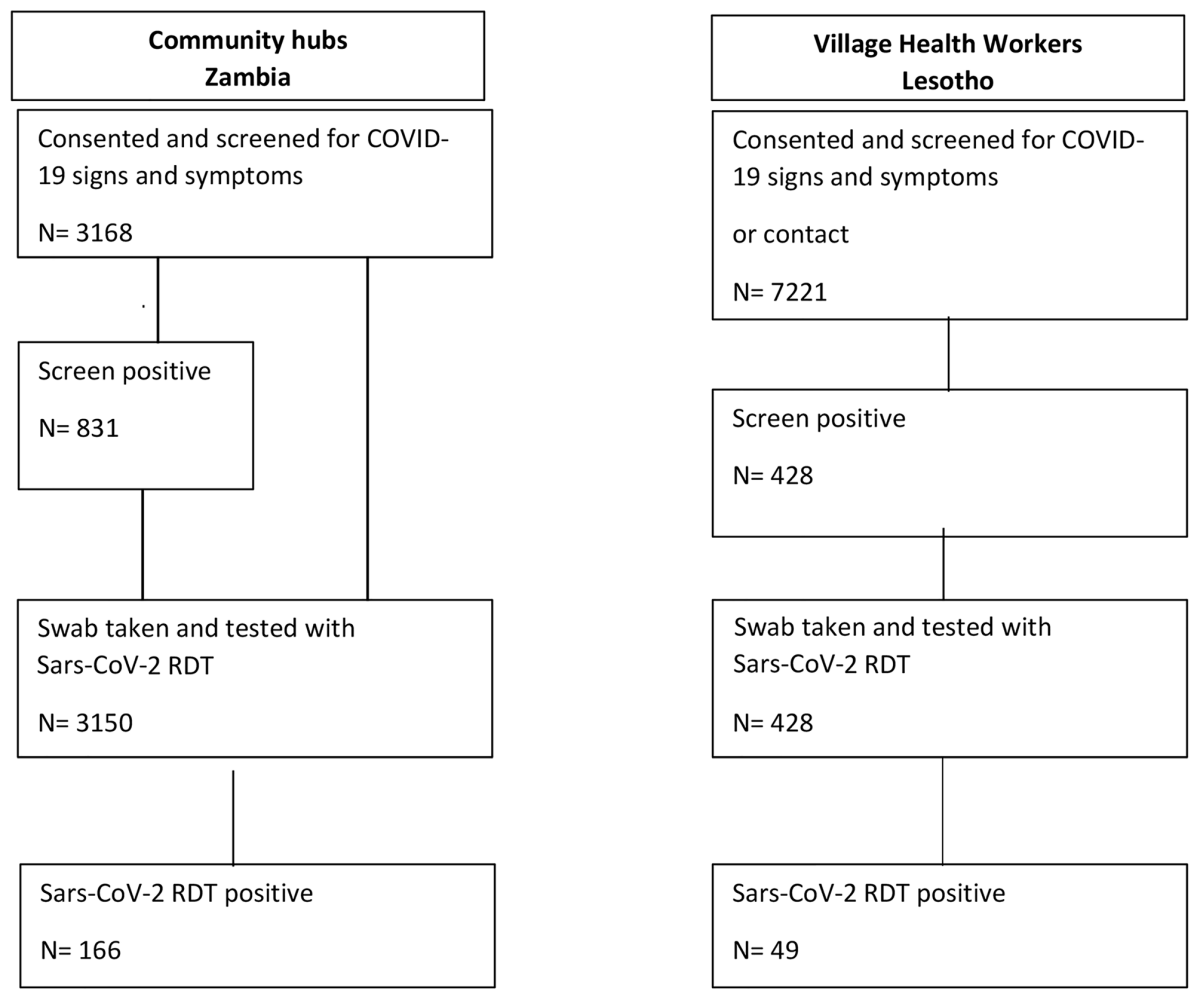
Flow diagram depicting screening, enrolment and case finding in the two community approaches

Figure 3 shows the flow diagram of enrolment for both community approaches. Under the VHW approach 7,221 people were screened of whom 428 (5.9%) screened positive for COVID-19 (symptoms and/or contact) and were enrolled for testing. In Zambia at the community hubs, a total of 3,168 persons consented and agreed to enrol, among these 831/3,168 (26.2%) screened positive (COVID-19 symptoms or household contact). In the hubs, 3150/3168 (99.4%) of those enrolled had a swab taken that was tested, while in Lesotho all the 428 enrolled had a swab taken and tested.

**Fig. 4:**
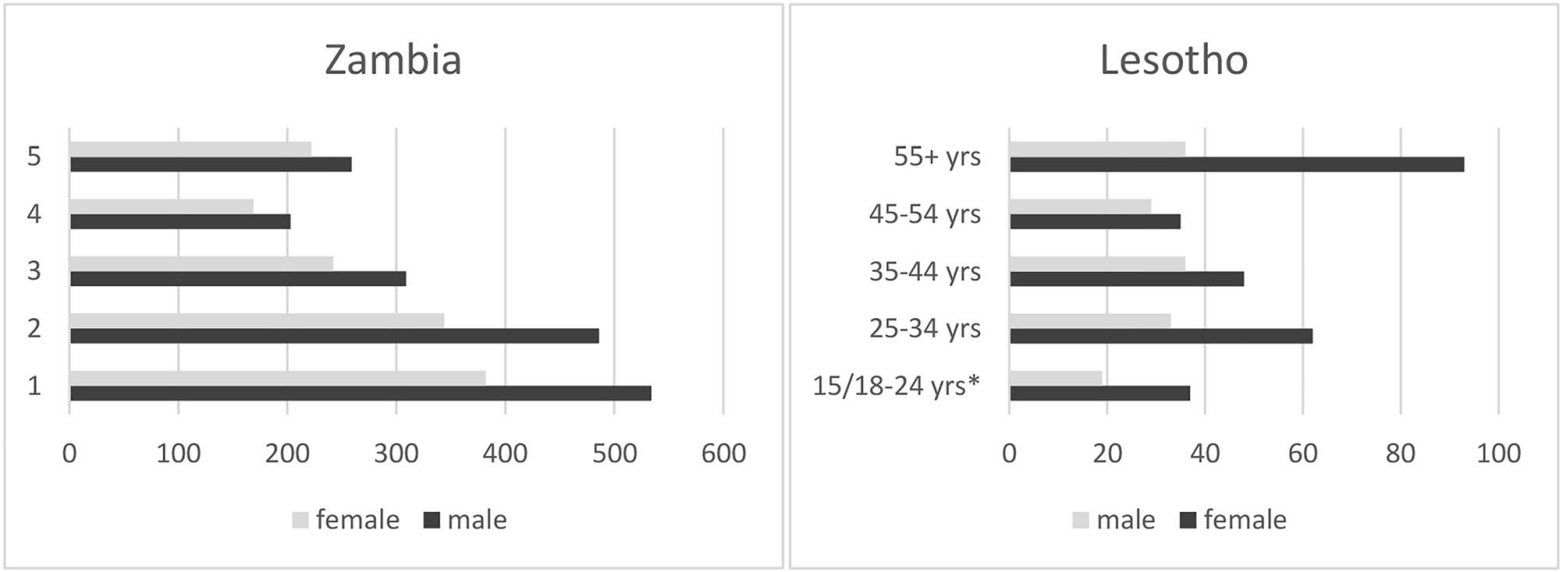
Age-sex distribution of those tested (n = 428) in the Village Health Worker approach in Lesotho (right) and those screened and tested (n = 3150) in the community hubs in Zambia (right)

Table 2 shows the characteristics of those tested in both approaches, 3150 persons in the community hubs and 428 in the VHW approach. In the VHW approach, 153 (35.7%) of persons tested were male while 275 (64.3%) were female. Close to a third of those enrolled (129/428 (30.1%)) were ≥ 55 years while 56/428 (13.1%) were 18–24-year-olds. In the community hubs, more males than females were tested, 1791 (56.9%) male versus 1359 (43.1%) female (Fig. 4; Table 2), overall age was 36.2 (SD 15.9) years old. More than half the participants tested, 1746/3150 (55.4%), were between 15 and 34 years old and 481/3150 (15.3%) were aged ≥ 55 years, while 916/3150 (29.1%) were 15–24 years. Age distribution was similar for male and females (Fig. 4).

**Table 2:** characteristics of people tested in the two approaches and those testing SARS-CoV-2 positive in Lesotho and Zambia

	overall	Community hubs - Zambia												Lesotho			
		all screened and tested at hub location				screened COVID-19 negative				screened COVID-19 positive				Village health workers			
		# Ag tested (n = 3150)	% among category	# SARS-CoV-2 positive	% SARS-CoV-2 positive	# tested	% among category	# SARS-CoV-2 positive	% SARS-CoV-2 positive	# tested	% among category	# SARS-CoV-2 positive	% SARS-CoV-2 positive	# tested (method)	% among category	# SARS-CoV-2 positive	% Ag positive
		**3150**	**100%**	**166**	**5.3%**	**2322**	**100%**	**23**	1.0%	**828**	**100%**	**143**	17.3%	**428**	100.0%	**49**	11.4%
**Age**	15/18–24 yrs*	**916**	**29.1%**	**44**	**4.8%**	661	28.5%	6	0.9%	**255**	30.8%	38	14.9%	**56**	13.1%	7	12.5%
	*25–34 yrs*	**830**	**26.3%**	**47**	**5.7%**	622	26.8%	9	1.4%	**208**	25.1%	38	18.3%	**95**	22.2%	13	13.7%
	*35–44 yrs*	**551**	**17.5%**	**39**	**7.1%**	406	17.5%	4	1.0%	**145**	17.5%	35	24.1%	**84**	19.6%	12	14.3%
	*45–54 yrs*	**372**	**11.8%**	**18**	**4.8%**	270	11.6%	4	1.5%	**102**	12.3%	14	13.7%	**64**	15.0%	6	9.4%
	*55 + yrs*	**481**	**15.3%**	**18**	**3.7%**	363	15.6%	0	0.0%	**118**	14.3%	18	15.3%	**129**	30.1%	11	8.5%
**Gender**	*Male*	**1791**	**56.9%**	**77**	**4.3%**	1361	58.6%	7	0.5%	**430**	51.9%	70	16.3%	**153**	35.7%	36	23.5%
	*Female*	**1359**	**43.1%**	**89**	**6.5%**	961	41.4%	16	1.7%	**398**	48.1%	73	18.3%	**275**	64.3%	13	4.7%
**TB Status**	Current /history of TB treatment*	**106**	**3.4%**	**6**	**5.7%**	52	2.2%	0	0.0%	***54***	6.5%	*6*	*11.1%*	***2***	0.5%	*1*	*50.0%*
	*no TB treatment history*	**3044**	**96.6%**	**160**	**5.3%**	2270	97.8%	23	1.0%	**774**	93.5%	137	17.7%	**426**	99.5%	49	11.5%
***COVID screen pos***	*yes*	***828***	**26.3%**	***143***	**17.3%**	*0*	0.0%	*n/a*	*n/a*	**828**	100.0%	143	*17.3%*	**428**	100.0%	428	*100.0%*
	*no*	***2322***	**73.7%**	***23***	**1.0%**	*2322*	100.0%	*23*	1.0%	**0**	0.0%	*n/a*	*n/a*	**0**	0.0%	0	*n/a*
***COVID symptomatic***	*yes*	***661***	**21.0%**	***138***	**20.9%**	*0*	0.0%	*n/a*	*n/a*	**661**	79.8%	138	*20.9%*	**260**	60.7%	*47*	*18.1%*
	*no*	***2489***	**79.0%**	***28***	**1.1%**	*2322*	100.0%	*23*	1.0%	**167**	20.2%	5	*3.0%*	**264**	61.7%	*0*	0.0%
***covid HH contact***	*yes*	***261***	**8.3%**	***37***	**14.2%**	*0*	0.0%	*n/a*	*n/a*	**261**	31.6%	37	*14.2%*	**99**	23.1%	*16*	*16.2%*
	*no*	***2889***	**91.7%**	***129***	**4.5%**	*2322*	100.0%	*23*	1.0%	**567**	68.5%	106	*18.7%*	**1**	0.2%	*0*	*0.0%*
	*unknown*	0	0	n/a	n/a	n/a	n/a	n/a	n/a	n/a	n/a	n/a	n/a	**324**	75.7%	*31*	*9.6%*
	*missing*	0	0	n/a	n/a	n/a	n/a	n/a	n/a	n/a	n/a	n/a	n/a	**4**	0.9%	*2*	50.0%

In Lesotho, 18.9% of persons enrolled reported having been tested for COVID-19 before. In the community hubs this information was not collected. In the VHW approach, 2 persons reported being currently on TB treatment. Information on a previous history of TB was not collected in Lesotho. From the people seen at the community hubs, 106/3150 (3.4%) reported a history of TB treatment including 7 who were on TB treatment when seen at the hubs.

#### SARS-COV2 case detection

In the two community approaches combined a total of 215 SARS-COV2 cases were identified. In the VHW approach, 49/438 (11.4%) persons tested positive for SARS-CoV-2., This was higher among males than females (36/153 (23.5%) males versus 13/275 (4.7%) females, p < 0.0001). Looking at the age distribution a larger proportion tested positive in the groups up to 44 years compared to those 45 years and older (13.6% versus 8.8% respectively, p = 0.06) with the highest proportion testing SARS-COV-2 positive observed in the 35-44-year-old at 14.3%. Patterns observed differed a bit in the community hubs. There, among 3,150 persons tested with Ag-RDT, 166 (5.3%) tested SARS-CoV-2 positive (Table 2). In Zambia, more females than males tested SARS-CoV-2 positive (6.5% versus 4.3% p = 0.0026), this pattern was the same among those screened positive for COVID-19 (18.3% versus 16.3%) as among those screened negative (1.7% versus 0.5%) for females and males respectively (Table 2). Looking at age, 7.1% of those 35–44 years investigated tested SARS-CoV-2 positive while this was 5.7% for those 25–34 years and less than 5% for all other age groups. The proportion testing SARS-CoV-2 positive in the age-group 35–44 years, was significantly higher at 7.1% than the proportion testing positive in all other age groups combined at 4.9% (p = 0.0182). A similar pattern was observed among those who screened positive for COVID-19, with 24.1% testing SARS-COV2 positive in the 35–44 year age group.

Though all attending the hubs were tested, most participants with a SARS-CoV-2 confirmed test were identified in those screening positive for COVID-19 (symptoms or household contact) at 17.3% (143/828) versus 1.0% (23/2322) (p < 0.0001) among those screening negative for COVID-19 (Table 2). This was more pronounced for those showing signs and symptoms (138/661 = 20.9%) than for those reporting having a household contact with confirmed COVID-19 (37/261 = 14.2%; p = 0.0097) (see Table 2).

**Fig. 5:**
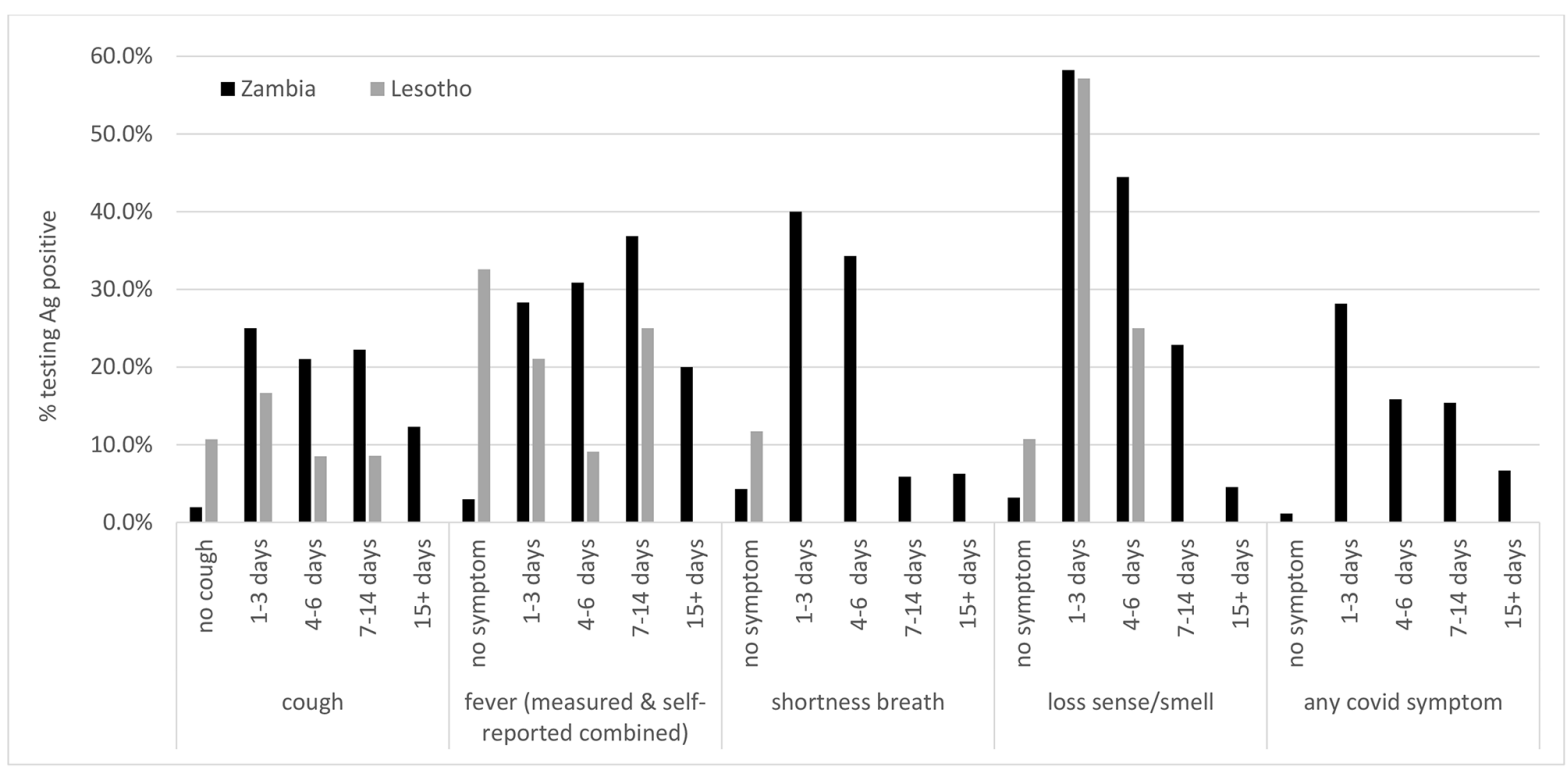
Proportion of antigen confirmed cases identified by their symptom reporting duration

Figure 5 outlines the percentage of those with antigen confirmed SARS-CoV-2 by the duration of their symptoms reported for both countries. It can be observed that those with (antigen) confirmed SARS-CoV-2 more often reported the COVID-19 suggestive symptoms for a short duration, i.e. 1–3 or 4–6 days. For example, of those reporting loss of sense/smell for 1–3 days, 60% tested antigen positive for SARS-CoV-2, this was similar for Lesotho and Zambia. Although for Lesotho the pattern was less pronounced, for example a third of SARS-COV-2 cases did not report any fever. More details are in supplementary table S1.

## Discussion

With two different approaches, the Village Health Workers approach, and the Community Hub approach, we were able to implement community-based COVID-19 screening and testing in Lesotho and Zambia. Overall, we managed to screen more than 10,000 people and identify a total of 215 antigen-confirmed SARS-CoV-2 cases within a 19-month period. The VHW approach attracted more females as compared to the community hub approach. The test positivity amongst participants who screened positive was 11.4% in the VHW approach in Lesotho and 17.3% in the hub approach in Zambia. The test positivity was highest in participants who reported a loss of smell and presented with symptoms for a short duration (1–3 days). In each approach, people accessed COVID-19 services within their communities at or close to their homes without having to travel long distances.

Differences observed in the two approaches could be related to differences in the strategies used for screening and testing. In the VHW approach, VHW visited households of participants with suspected SARS-CoV-2 infection while in the community hubs, community health workers waited for community participants at a designated place in the community. The difference in the type of population attracted by each approach may be explained by the fact that women are more likely to be found around the house because of child-care and household duties as has been found in other studies [14]. In Lesotho the symptom screening algorithm was wider than in Zambia and testing was restricted to those screening positive for signs and symptoms or being a COVID-19 contact while in the hubs all attending were offered SARS-COV-2 testing.

In other studies, an even higher number of people could be reached, and the yield was higher than what we observed in our study. A study in South Africa [1], covering all the South African provinces, reported a daily rate of 10,000 to 15,000 COVID-19 tests with a positivity rate of 19.25%. The difference observed with the findings in our study may be due to differences in the number of people targeted, the number of human resources deployed and the COVID-19 prevalence. The community screening and testing strategy in this South African study, was implemented countrywide with more than 28,000 community health workers while we targeted a more focused area including a smaller number of people and deployed a lower number of screeners and testers. Another African study in the Democratic Republic of the Congo (DRC) deployed a community-based testing strategy showing increased access resulting in improved case detection and response [15]. Both studies had a much larger scale than our study, the South Africa study was a national system being implemented while the DRC study was conducted in 9/26 provinces of the country. Both studies reported challenges with staffing (use of highly qualified people: laboratory technicians, epidemiologists, nurses etc.) and logistics (transport, travel for long distance). Our study demonstrates that with less qualified staff, a COVID-19 community-based screening and testing program can be implemented (task shifting). In both the South African and the DRC study, testing and specifically taking of the nasopharyngeal swab was done by a highly qualified cadre of staff while in our study, after a short training, community health workers could correctly collect nasopharyngeal swabs and perform SARS-CoV-2 testing. Also, in our study, logistics was not a big challenge as VHWs served their own community and community hubs were implemented nearby health clinics. Also, our approaches could be used for the implementation of complex health programs (community case management for specific conditions etc.)

The difference in SARS-CoV-2 positivity yield among people that were tested may be due to the prevalence of COVID-19 in the population tested. Since the beginning of the pandemic, South Africa reported much higher numbers of COVID-19 cases [16] than Lesotho and Zambia where our two community strategies were implemented. However, this and other studies in South Africa [1, 17, 18] reporting on community-based COVID-19 screening and testing did not report much detail on characteristics of participants seen and/or tested but rather reported aggregated data. Therefore, it is difficult to compare such details with the participants included in our study.

We observed that in Lesotho the proportion of males testing SARS-CoV-2 positive was much higher than that for females (23.5% versus 4.7%), while in Zambia the reverse was observed, with yield of SARS-CoV-2 slightly higher among females than males (6.5% versus 4.3% respectively). Data compiled by sex, gender of the COVID-19 Project [19] found similar patterns to what we observed in both countries [20, 21]. Existing literature does show a gender imbalance for SARS-CoV-2 with regards to mortality, reporting higher case fatality rates in males compared to females in many countries [19, 22–24]. Biological and behavioural reasons are believed to be behind reported differences by gender [22]. We have no explanation for the observed difference by gender in proportion testing SARS-CoV-2 between the two countries.

Our work contributed to the evidence base on community-based testing strategies in the African setting. The fact that our study was conducted (partly) in rural African communities with challenges to access to basic health services and where community-based COVID-19 screening and testing are mostly needed is an added value. Community based testing can help scale up SARS-CoV-2 testing and improve case-detection rates in the community as also concluded by Otshudiema and colleagues [15].

Our study has various limitations. Firstly, we implemented this study in a small number of communities including a relatively low number of people. Replicating this study in a larger population and more communities may come with additional challenges that we did not experience during the implementation of our two approaches in Lesotho and Zambia. Secondly, the analysis done in this study was mainly descriptive and both countries implemented a different strategy at a different time. The number of cases identified varied, and testing took place in different epidemic waves and as COVID-19 is a rapid disease no direct comparison could be made.

In conclusion, in this project we learned that implementing SARS-COV2 screening and testing by lay health workers in the community is feasible and people do come forward for testing. Characteristics of the population screened, tested, and identified to have SARS-CoV-2 are described to help guide development of future testing strategies. Offering testing in the community is preferable and if resources (staff and/or consumables) are scarce testing could be targeted at those with signs/symptoms of SARS-COV2.

## Electronic supplementary material

Below is the link to the electronic supplementary material.

Supplementary Material 1

## Data Availability

The datasets used and/or analysed during the current study are available from the corresponding author on reasonable request.
