# Supplementary material for: Creating access to SARS-CoV-2 screening and testing through community-based COVID-19 case-finding, observations from cross-sectional studies in Lesotho and Zambia
Source: BMC Public Health. 2023 Jul 24;23:1414. doi: 10.1186/s12889-023-16306-2 (PMC10364377; doi:10.1186/s12889-023-16306-2)
Supplement: Supplementary file 1 — Supplementary Material 1 [file 12889_2023_16306_MOESM1_ESM.pdf]

Supplementary table S1. Reporting of symptoms and their duration for all participants tested for SARS-COV-2 antigen and those with confirmed SARS-COV-2 (by antigen testing).

		Community hubs				Village HW			
Symptom reporting		number tested		SARS-COV2- cases (Ag)		number tested		SARS-COV2- cases (Ag)	
		n=3150		n=166		n=428		n=49	
		# Ag tested (n=3150)	column % among those reporting the symptom	n	%	n	column % among those reporting the symptom	n	%
	overall	3150		166	5.3%	428		49	11.4%
cough	*no cough*	2620		51	1.9%	243			
	1-3 days	184	34.6%	46	25.0%	96	53.3%	16	16.7
	4-6 days	157	29.5%	33	21.0%	47	26.1%	4	8.5
	7-14 days	126	23.7%	28	22.2%	35	19.4%	3	8.5
	15+ days	65	12.2%	8	12.3%	2	1.1%	0	0.0
fever (measured & self-reported combined)	*no symptom*	2889		86	3.0%	132			
	1-3 days	113	43.3%	32	28.3%	19	55.9%	4	21.1
	4-6 days	81	31.0%	25	30.9%	11	32.4%	1	9.1
	7-14 days	57	21.8%	21	36.8%	4	11.8%	1	25
	15+ days	10	3.8%	2	20.0%		0.0%	0	0.0
shortness breath*	*no symptom*	3026		130	4.3%	418			
	1-3 days	55	44.7%	22	40.0%	5	83.3%	0	0.0
	4-6 days	35	28.5%	12	34.3%	1	16.7%	0	0.0
	7-14 days	17	13.8%	1	5.9%		0.0%	0	0.0
	15+ days	16	13.0%	1	6.3%		0.0%	0	0.0
loss sense/smell	*no symptom*	2979		95	3.2%	410			
	1-3 days	79	45.9%	46	58.2%	7	50.0%	4	57.1
	4-6 days	36	15.3%	16	44.4%	4	28.6%	1	25
	7-14 days	35	14.9%	8	22.9%	3	21.4%	0	0.0
	15+ days	22	9.4%	1	4.5%		0.0%	0	0.0
any covid symptom	*no symptom*	2481		28	1.1%				
	1-3 days	309	45.9%	87	28.2%				
	4-6 days	183	15.3%	29	15.8%				
	7-14 days	117	14.9%	18	15.4%				
	15+ days	60	9.4%	4	6.7%				
